# Promoting inclusivity and partnership with recipients of health services: Short report on redesigning Engaging All Voices (previously Consumer Voice)

**DOI:** 10.21203/rs.3.rs-9224470/v1

**Published:** 2026-04-23

**Authors:** Eva N. Woodward, Irenia A. Ball, JoAnn E. Kirchner, Sara J. Landes, Leslie R. M. Hausmann, Cathleen Willging

**Affiliations:** University of Arkansas for Medical Sciences; Central Arkansas Veterans Healthcare System; University of Arkansas for Medical Sciences; University of Arkansas for Medical Sciences; VA Pittsburgh Healthcare System; Pacific Institute for Research and Evaluation

**Keywords:** service users, consumer, patient engagement3, patient and public involvement, community engagement, implementation science, quality improvement

## Abstract

**Background:**

Researchers identified “preparing patients to be active in innovations” as one of the top five most effective implementation strategies; this included educating patients to be engaged in their care. If engaging patients in their own care enhances adoption of innovations, engaging patients and service users across any setting (e.g., healthcare, education, carceral) in implementation activities may also enhance adoption. To provide guidance on this topic, we developed Consumer Voice: a free, online platform for community engagement in implementation efforts. In July 2023, we disseminated the tools and received feedback that “consumer” had a negative connotation for some. This feedback mimicked discontent with terminology for the “service user” in the broader implementation field. Our study goal was to identify inclusive, acceptable terminology for the “service user” in implementation practice or research.

**Methods:**

We collected quantitative data then qualitative data from service users, implementers, and implementation researchers in two phases: 1) Brainstorming and prioritizing terminology for service users through Nominal Group Technique, then 2) Contextualizing implications of different terms through focus groups. Between phases, we used quantitative counts to inform sampling and inductive qualitative analysis.

**Results:**

Participants represented a range of demographic groups; over 40% identified primarily as individuals receiving health services, 30% as implementers, and 26% as implementation researchers. There was consensus to rename our engagement tools “Engaging All Voices.” Our data indicated there was no universally acceptable term for “service user.” Even within similar communities, some terms were controversial e.g., “patient,” “peer.” Two terms felt off-limits to most: “stakeholder” and “user.” Participants thought any engagement language should convey a sense of action and partnership to move beyond performative, superficial “engagement.”

**Conclusions:**

We sampled people from multiple service settings and thus, findings may generalize in healthcare, education, carceral, and other systems. Our experience redesigning Engaging All Voices revealed that how recipients of innovations are referred to can shape receptiveness to engagement approaches. There is no single “right” term to refer to service users across settings. Engaging All Voices now guides learners in an early exercise to determine terminology preferred by service users throughout an implementation effort.

## Introduction

Researchers have identified “preparing patients to be active in innovations” as one of the top five most effective implementation strategies to date.^1-3^ Preparing patients to be active includes educating them to be engaged in their care by asking questions about care guidelines and rationale for clinical decisions. These data^1-3^ provide a scientific signal that engaging patients in the organizational “inner workings” to design and deploy health services, versus only engaging them in their care, could improve adoption of effective treatments.^1,4^

There has been little guidance on what preparing service users of an innovation entails or how to co-develop and deploy strategies with the service user, who we define as the intended recipients of an innovation—those expected to receive and benefit from a service. Service user engagement can range in level of intensity from one-time interactions to ongoing efforts. Roles for service users who receive health services in any setting (e.g., schools, clinics) in implementation activities may include guiding cultural adaptation of a treatment before launch in a new setting, advising on implementation strategy selection, or serving as an implementation practitioner.^4^

In 2018, we conducted an environmental scan documenting the need for guidance to engage service users in implementing services that are meant for them.^5^ Following Kwan et al.’s Fit-to-Context framework for dissemination,^6^ we attempted to fill this need by creating tools for implementers to engage service users in implementation efforts. We involved patients in a health system *and* professionals from multiple systems (e.g., hospital, criminal justice, community clinics, schools) in codesigning and user-testing these tools.^7^ The result was Consumer Voice: a free, online platform offering tools for community engagement in implementation efforts.

In July 2023, we disseminated Consumer Voice tools publicly for implementers. We received feedback from potential users that the word “consumer” had a negative connotation in social sciences and public health because it drew from business and for-profit fields, and that some service users did not want to be referred to as consumers in healthcare settings. We needed to enhance the “product to context fit” of the engagement tools.^6^ This feedback also mimicked lack of consensus with terminology for the “service user” in the broader implementation research and practice field.^8,9^ Our study goal was to identify inclusive, acceptable terminology referring to the “service user” and its potential consequences to a broad variety of social positions we encounter in implementation practice and research. This study was also a next step to enhance product-context fit to a variety of cultural norms in which our engagement tools may be used, consistent with Kwan et al’s framework.^6^

## Methods

### Study Design

Using an exploratory sequential design,^11^ we collected data from people involved in implementation processes in two phases: 1) brainstorming and prioritizing terminology when referring to service users through Nominal Group Technique, and 2) contextualizing implications of applying different terms through focus groups (Table 1). Study activities were deemed human subjects research by the Central Arkansas Veterans Healthcare System Institutional Review Board. We conducted a verbal and written informed consent process.

### Participants in Nominal Group Technique and Focus Groups

We recruited from multiple service settings including educational, healthcare, and other places where health services are delivered. From April 2024 to September 2025, we sent e-mails to recruit participants from our team’s North American networks in implementation science, practice, and community organizations (e.g., patient/family advisory councils for health systems, national learning collaborative for practice facilitators). Due to funding regulations, we offered service users of the U.S. Veterans Health Administration received a $50 check; other participants received a $50 Amazon gift card.

#### Service users.

These were people over 18 years old receiving health services (e.g., patients) because we wanted their perspectives reflected in the nomenclature.

#### Implementers.

Implementers were professionals responsible for navigating the launch / quality of a health service in any setting. Our purpose was to ensure that our engagement tools were attractive to them.

#### Researchers.

These researchers were participants, not our research team. They were recruited based on implementation science or community engagement experience to learn their perspectives.

### Data Collection and Analysis between Phases

#### Phase 1: Brainstorming and prioritizing.

We hosted 50-minute virtual meetings using Nominal Group Technique^13^ to brainstorm then prioritize preferred names for service users and our engagement tools across different settings. Each group consisted of either service users, implementers, or researchers and 1–2 people from the contact organization who co-facilitated the meeting. Thus, most groups consisted of participants on the same “level” regarding power in decision making about implementing a health service. Each person was sampled only once.

Our goal was to synthesize a set of preferred terms for “service user” across settings. We asked these questions with five minutes to silently brainstorm: “What are potential names for the person receiving services besides ‘consumer?’” and “What would you suggest we rename ‘Consumer Voice?’” We asked each participant to share all their responses verbally and visually posted all ideas on a virtual whiteboard all could see. Next, we prompted participants to discuss rationales for their ideas for 5–10 minutes. Finally, each participant was given a specific number of votes based on the list of brainstormed choices; the longer the list, the more votes were allowed (*Range* = 3–10).^14^ Participants could spread those votes across one or more terms they preferred. We repeated this process with six different groups until we heard the same set of options. One researcher facilitated while another took notes and transcribed video recordings (see reflexivity statement).

##### Phase 1 data analysis:

We summed votes across Phase 1 meetings to rank the top five preferred names for the “service user” and top three alternate names for the engagement tools. We shared these results alongside ideas that were mentioned more than once during participants’ discussions with our entire research team to interpret findings. We analyzed participants’ demographics after Phase 1. For Phase 2, we recruited populations underrepresented in Phase 1 to maximize participant variation.^12^

#### Phase 2: Contextualizing.

We recruited a new sample for four focus groups to develop consensus on a new name for our engagement tools and discuss unintended or intended consequences of preferred terms for “service users.” This is a form of member checking to enhance validity by allowing participants to broaden, clarify, or generate new information based on Phase 1 data.^15^ We shared a low-fidelity prototype advertisement for the engagement tools and elicited participants’ reactions to it, including proposed names of the tools. Sample focus group questions were: “For the name of tools on the advertisement, what does this word make you think about?” and “What pros or cons do you see to using this word?” By the last focus group, we did not hear any new information, and ceased sampling.^16^

##### Phase 2 qualitative analysis:

Both researchers (ENW and IAB) completed a verbal and written debriefing process using memos, checked video recordings for unclear areas, and completed a summary of written data from each focus group. They organized written data into a single matrix, with questions posed during focus groups as column headers and participant’s primary role (service user, implementer, researcher) in each row. They added to the cells all repeating ideas from participant discussions during Phase 1 sessions and Phase 2 focus groups.

The goal of our inductive qualitative analysis was to generate major findings regarding: “*What are recommendations for language for the ‘service users’ in implementation efforts?”* and *“What is an ideal name for the engagement tools?”* Each analyst independently reviewed the matrix, combining concepts described more than once into repeating ideas, then synthesizing them into higher-level concepts. Analysts met and compared initial analyses, referring to the data to verify, clarify, and expand. They came to consensus on a potential name for engagement tools—Engaging All Voices.

#### Final Data Validation.

We sent a one-question e-mail survey to all participants comparing an older advertisement for Consumer Voice to a new advertisement for Engaging All Voices. We asked, “W*hich flyer resonates more with you?”* The Engaging All Voices flyer was chosen by 87.5% of participants (21 out of 24).

## Results

### Demographics

Table 2 reflects a diverse and knowledgeable sample, grounding results in relevance and inclusiveness. Over 40% identified as individuals receiving health services. Many participants (36%) reported some familiarity with community engagement, enhancing depth of their feedback.

### Quantitative Findings from Nominal Group Technique

The terms participants prioritized are in Table 3. During analysis interpreting Phase 1 results, our team noted that many of these words would not fit every implementation setting (e.g., ‘client’ not suitable in schools). Also listed were participants’ top names for our engagement tools. The majority voted for suggestions that were either very specific or for a format substituting the original “consumer” for another noun referring to service users (e.g., Community Voice).

### Qualitative Findings from Focus Groups

Participants in the last two focus groups found unanimous consensus to rename our engagement tools “Engaging All Voices.” Our qualitative data indicated there was no universally accepted term for a service’s end user. Four findings are presented with repeating ideas below.

“Engaging All Voices” may not be completely clear on its own, but it does have appeal, accurately applies to a broad audience, and evokes inclusivity. One implementer said, “*With a [more generic name], you have to do a little work to think about ‘am I included in this’?”* The broad name was acceptable if accompanied by clear, compelling messages that elaborated on the tools’ concept, functions, and intended audience (e.g., tagline). Also, more specific names did not accurately represent the tools’ generalizability across populations (e.g., Student Voice). Some implementers wanted more workforce-centered language in the tools, including one who said, *“‘Workforce voices’ don’t seem to be included in [the ads for the tools] even though the ultimate service users are people in the workforce, so that omission could be confusing.”*

A second finding was that some terms were controversial even within communities with shared demographic characteristics, such as “patient,” “peers,” “people in need,” and “consumer.” Within a focus group of people who access mental healthcare at the same clinic, two participants with a business/finance background felt comfortable with “consumer” while two other participants rejected the term because it implied a false sense of choice in seeking health services. Some participants did not like “patient” because it felt disempowering. Two terms felt off-limits to most: “stakeholder” and “user.” Although “stakeholder” was familiar to implementers, it made them think about executive leaders, or as one implementer shared, “*People who have power, money, make decisions and not all people we serve… would be that.”* The word “user” connoted negativity, as one implementer said it implied people are “*substance users, potentially, or “users” of a health system in a negative way.*”

A sense of action and partnership should be conveyed through the words used in engagement to evoke a motivated and committed group of personnel ready to move beyond performative, superficial “engagement.” Specifically, language about “voices” was appealing in the tools. Service users expressed that, “*advocacy is important, so ‘voice’ is important”* because they wanted to be empowered through having their voices heard and respected. Implementers and service users preferred active verbs to describe service users’ engagement. One service user said, “*I like to think of ‘engage’ as it is more active than ‘including;’ We are supposed to be working together.”* This highlighted the concept of collaboration.

Participants responded positively to the tools’ purpose to promote inclusivity and liked that the tools offered workforce personnel actions to move toward inclusivity. Most participants perceived that the tools would teach them something new. Meaningful partnerships were important to participants, and they felt tools that help personnel do that are needed. As one implementer stated, “*It would help me do my job better – means that efforts we are implementing, improvement efforts, that are not just top-down mandates, but they can be two-way streets, working in partnership together to be the best it can be.”*

## Discussion

There was no consensus on acceptable terms for the service user in the context of implementation efforts across any setting. Language that promoted inclusivity and implied partnership was more important than selecting a single label for service users. The lack of consensus across diverse participants from several social positions validates that we, as implementers and researchers, also struggle with these terms.

As a result, we added a learning objective to Engaging All Voices to co-develop terminology that is culturally responsive to people meant to receive the service. Specifically, there is a module in the toolbox entitled “Prepare to Work with Everyone” which highlights tasks to be accomplished early in an implementation partnership, such as identify why it is important to engage others in a particular effort and plan ways to compensate others for their contributions. We do not recommend any certain terminology. We propose activities to determine preferred terms defined with the community in question.

One issue in implementation science is that we may not critically examine how people involved in implementation efforts respond to terms used “about” them rather than “by” them. One result of our study was that common terms in implementation science and practice, such as “patient,” “peer,” “people in need,” and “consumers,” elicited mixed reactions. Even among similar groups, participants disagreed on terms, with some suggesting their use because they were clear and others recommending against their use because they implied a state of disempowerment or inaccuracy. A common word in our field, “stakeholder,”^17-19^ was perceived negatively by service users and implementers. We might avoid using “stakeholder” unless it is the most accurate *and acceptable* term. Because implementation science and practice rely on significant interpersonal collaboration,^20-22^ it behooves us to be thoughtful about our language about people we collaborate with. In future implementation efforts, we may need to conduct an assessment to determine acceptable terminology for service users.

Implementation science paradigms, influenced by context, often stress urgency, efficiency, and rapid knowledge production. yet, our rapid knowledge translation can sacrifice relatability, relationship-building, and trust among our collaborators. Processes to “design for dissemination” that harness community-engaged implementation, as in our study, can explore this tension.^6^ Establishing trust among all people involved in an implementation effort is increasingly seen as vital to an innovation’s success.^21^

## Conclusion

We identified a new name for our engagement tools: Engaging All Voices. This compendium includes free tools to help implementers engage service users in launching and sustaining health services. It is hosted on two platforms: one within the Veterans Health Administration server,^23^ and one for individuals outside the Veterans Health Administration.^24^

Engaging people who will receive health services is a potentially powerful but underutilized implementation strategy. Redesigning Engaging All Voices revealed that how recipients of innovations are referred to can shape receptiveness to engagement. Our study suggests there is no “right” term for service users across any setting and provides a process by which implementers can thoughtfully engage service users to determine suitable terminology. Future work should evaluate Engaging All Voices feasibility and outcomes.

## Figures and Tables

**Table 1:** Study matrix showcasing multiple methods

Procedures	Sample (n = individual participants)	Goal	Analysis or interpretation
Phase 1: Nominal Group Technique (n = 6 sessions)	Service users (n = 47) Implementers (n = 5) Researchers (n = 6)	Generate ideas and vote on terminology priorities for “service users.”	Compile quantitative voting sums and descriptive statistics of participant demographics.
Connect data to inform qualitative focus group questions and determine participant sampling strategy.	Panel of community engagement and implementation researchers (n = 4, coauthors)	Align the list of top preferred terms from Nominal Group Technique sessions to open-ended questions to contextualize terms in focus groups.	Vote to narrow the potential list to the top three choices and identify participant groups that need representation in the next phase.
Phase 2: Focus groups (n = 4 groups)	New sample of service users (n = 7), implementers (n = 12), Researchers (n = 2)	Conduct member checking to contextualize and understand the implications of adopting terms	Perform rapid qualitative analysis using a template approach.
Make meta-inferences	Same panel of community engagement and implementation researchers (n = 4, coauthors)	Determine consensus on top-rated terms and their contextual implications.	Clarify the new name for Consumer Voice tools upon refinement of results.

**Table 2:** Participant Demographics

Demographic Characteristic	N (%) *
**Age**	*Mean* = 49.7 years, *Range* = 26–78
**Roles (check all that apply)**	Veteran of the US military*
Veteran of the US military*	20 (27%)
Service user of healthcare	31 (42%)
Facilitator, quality improvement person, clinic manager	22 (30%)
Implementation researcher	19 (26%)
Caregiver and other	13 (12%)
**Gender Identity** *	
Man	21 (29%)
Woman	50 (68%)
**Education Level**	
College degree or greater	28 (58%)
No college degree	20 (42%)
**Racial Identity**	
American Indian or Alaska Native	10 (13%)
Black or African American	28 (38%)
White	26 (35%)
**Ethnicity**	
Non-Hispanic, Latino, or of Spanish origin	60 (82%)
Hispanic, Latino, or of Spanish origin	11 (20%)
Ancestors from Puerto Rico	10 (14%)
**Sexual Identity**	
Straight, that is, not gay or lesbian, etc.	56 (76%)
**Geographic Location based on zip code** *	
Urban	34 (65%)
Rural	14 (26%)

* Note: Counts < 10 participants are not depicted to prevent re-identifying them. We asked about U.S. military Veteran status as this is our health system. Not all percentages = 100 because some participants endorsed “prefer not to answer” to some questions. Gender identity: some participants identified as non-binary. Racial identity: Some participants identified also as Asian (e.g., Chinese, Thai) or “another race.” Ethnicity: Some had as ancestors from another place of Hispanic, Latino, or Spanish origin. Sexual identity: Some identified as bisexual, lesbian, gay, or another identity. Zip code translated to Rural Urban Commuting Area code: 1–3 = urban, 4–9 = rural, 10 = highly rural. Some participants were outside the U.S.

**Table 3:** Terms and Engagement Tool Names Prioritized by Participants

Top Five Terms Prioritized for the Service User	Count^a^
Client	45
Participant	32
Constituent	21
Patient	19
Survivor	19
Top Three Names for Engagement Tools	Count^b^
Specific Option (e.g., Guiding Light, Raising the Bar)	15
[Placeholder Noun] Voice (e.g., Client Voice)	13
[Placeholder Verb] Voices (e.g., Empower Voices)	4
Grand Total of Choices	32

Note.

A. Votes will exceed the number of participants.

B. Votes do not reflect all participants, as 50%of the Nominal Group Technique participants did not have time to vote on their brainstormed terms.

## Data Availability

The datasets generated and analyzed for this study are not listed in a public repository. Please contact the first author to discuss use of de-identified datasets.
